# Health Care Professionals’ Experiences of Web-Based Symptom Checkers for Triage: Cross-sectional Survey Study

**DOI:** 10.2196/33505

**Published:** 2022-05-05

**Authors:** Sari Kujala, Iiris Hörhammer

**Affiliations:** 1 Department of Computer Science Aalto University Espoo Finland; 2 Department of Industrial Engineering and Management Aalto University Espoo Finland

**Keywords:** adoption, symptom checker, triage, health care professional, survey, online health, digital health, health organizations, health care

## Abstract

**Background:**

Web-based symptom checkers are promising tools that provide help to patients seeking guidance on health problems. Many health organizations have started using them to enhance triage. Patients use the symptom checker to report their symptoms online and submit the report to the health care center through the system. Health care professionals (registered nurse, practical nurse, general physician, physiotherapist, etc) receive patient inquiries with urgency rating, decide on actions to be taken, and communicate these to the patients. The success of the adoption, however, depends on whether the tools can efficiently support health care professionals’ workflow and achieve their support.

**Objective:**

This study explores the factors influencing health care professionals’ support for a web-based symptom checker for triage.

**Methods:**

Data were collected through a web-based survey of 639 health care professionals using either of the two most used web-based symptom checkers in the Finnish public primary care. Linear regression models were fitted to study the associations between the study variables and health care professionals’ support for the symptom checkers. In addition, the health care professionals’ comments collected via survey were qualitatively analyzed to elicit additional insights about the benefits and challenges of the clinical use of symptom checkers.

**Results:**

Results show that the perceived beneficial influence of the symptom checkers on health care professionals’ work and the perceived usability of the tools were positively associated with professionals’ support. The perceived benefits to patients and organizational support for use were positively associated, and threat to professionals’ autonomy was negatively associated with health care professionals’ support. These associations were, however, not independent of other factors included in the models. The influences on professionals’ work were both positive and negative; the tools streamlined work by providing preliminary information on patients and reduced the number of phone calls, but they also created extra work as the professionals needed to call patients and ask clarifying questions. Managing time between the use of symptom checkers and other tasks was also challenging. Meanwhile, according to health care professionals’ experience, the symptom checkers benefited patients as they received help quickly with a lower threshold for care.

**Conclusions:**

The efficient use of symptom checkers for triage requires usable solutions that support health care professionals’ work. High-quality information about the patients’ conditions and an efficient way of communicating with patients are needed. Using a new eHealth tool also requires that health organizations and teams reorganize their workflows and work distributions to support clinical processes.

## Introduction

### Background

Web-based symptom checkers are promising tools that provide help to patients seeking guidance on health problems [1]. Algorithm-assisted symptom checkers ask patients questions about their symptoms and may provide them with potential diagnoses, inform them about the urgency of seeking care, and direct them to appropriate care settings [2].

Many health organizations have started using symptom checkers to guide patients to the most appropriate course of action [2-5]. Notably, evidence of the diagnostic accuracy and impacts of web-based symptom checkers remains scarce [1], but they may supplement resource-intensive telephone triage lines common in primary care [2]. Recently, the COVID-19 pandemic has created pressure to use web-based symptom checkers to avoid face-to-face contact and preserve urgent care capacity [6,7].

Patients are generally highly satisfied with symptom checkers [1]. In a survey study involving the Isabel Symptom Checker, most patients perceived it to be useful for diagnosis [8]. They often used the symptom checker to better understand the reason for their symptoms and to decide whether to seek in-person health care. Users of the Erdusyk Symptom Checker also acknowledged its usefulness to avoid unnecessary visits to general practitioners [3].

However, the impact of symptom checkers on the urgent care system and the wider scope of health care is not known [1]. For example, triage advice is generally risk averse, encouraging patients to seek care for conditions in which self-care would be more appropriate [2]. In a recent study, only 5 out of 15 symptom checkers were found to be superior to the accuracy of laypersons, and the services were suspected to increase resource use in health care [9]. Moreover, symptom checkers are most often used by younger, more educated, and female patients [1,6].

As technology develops, the performance of symptom checkers can be expected to improve [2]. However, their success depends on health care professionals’ acceptance and the influence of symptom checkers on their workflow [10]. Health care professionals also influence patients’ adoption of eHealth services with their endorsement [11,12].

The goal of this survey study is to identify health care professionals’ experiences of symptom checker use in triage and analyze factors influencing their support for these tools. The two examined symptom checkers are used to help patients in obtaining appropriate care, but only one of them provides the patient with immediate information on conditions that correspond to their symptoms. Patients report their symptoms online and submit the report to the health care center through the symptom checker. Health care professionals (registered nurse, practical nurse, general physician, physiotherapist, etc) receive patient inquiries with urgency rating, decide on actions to be taken, and communicate these to the patients. The findings provide a better understanding of how symptom checkers can support efficient clinical work. Sustainable use of eHealth tools requires engagement of both professionals and patients [13]. Therefore, we also study whether professionals’ support is manifested in their interactions with patients.

### Research Model

The research model is based on the previous version that was tested in the preimplementation phase of a patient portal [14]. Similar to what was found in the preimplementation phase, we hypothesize the following notions after implementation, when the symptom checkers are used in health care organizations:

Hypothesis 1: the perceived usability of the symptom checker is positively associated with health care professionals’ support for the symptom checker.

Hypothesis 2: the positive influences on the work are positively associated with health care professionals’ support for a symptom checker.

Hypothesis 3: the positive influences on patients are positively associated with health care professionals’ support for a symptom checker.

Hypothesis 4: the perceived threat to professional autonomy is negatively associated with health care professionals’ support for a symptom checker.

In the preimplementation phase, expectations of good implementation practices in the work unit were positively associated with professionals’ support [14]. Accordingly, we hypothesize the following:

Hypothesis 5: organizational support for the use of the symptom checker is positively associated with professionals’ support for a symptom checker.

Professionals’ endorsement of eHealth tools is crucial in a patient’s decision to adopt health technology [15]. To test whether professionals’ support is manifested in their interactions with the patients, we hypothesize the following:

Hypothesis 6: health care professionals’ support for a symptom checker is positively associated with professionals’ promotion of the tool to the patients.

Hypothesis 7: health care professionals’ support for a symptom checker is positively associated with their provision of instruction to patients on the use of the tool.

## Methods

This study was designed as a cross-sectional survey of the health care professionals using symptom checkers for triage in Finland. A web-based questionnaire was developed to identify the experiences of health care professionals, including nurses, physiotherapists, and physicians.

### Study Setting

Both web-based symptom checkers studied have been adopted in Finnish public primary care. They are used to help patients in obtaining appropriate care. Inquiries coming into primary care can be digitally managed through the services instead of traditional phone-based triage. Patients report their symptoms online, and if they wish, they submit a report to health centers. The symptom checkers assess the urgency of care, and, if needed, guide patients to contact emergency care. Health care professionals (usually nurses) receive a list of inquiries with urgency rating to decide on actions to be taken and communicate these to the patients. Professionals may also inquire further information from patients or forward the task to a physician or another professional if needed. Detailed information on the symptom checkers following the guideline extension for evaluation of interventions with an artificial intelligence component [16] is in Multimedia Appendix 1.

In the first symptom checker, Omaolo oirearvio, the patient first chooses a symptom checker that best describes their condition, such as low-back pain, urinary tract infection, or upper respiratory tract infection. In March 2020, the number of the symptom checkers had increased to 16, including a generic symptom checker that is not specific to any certain symptoms. While diagnosis can only be made by a medical doctor in Finland, Omaolo oirearvio provides information on health problems that correspond to the patient-reported symptoms and recommendations for self-care. Professionals evaluate the patient’s inquiry by reading a provided summary of the symptoms, relate them to the patient’s medical records, and contact the patient using the messaging functionality. Unlike the other symptom checkers, the triage process of the urinary tract infection and COVID-19 symptom checkers can be entirely digitalized. A patient can reserve a COVID-19 test or reserve a prescription for urinary tract infection using the symptom checkers.

Omaolo oirearvio has been evaluated to be easy to use and understandable to most patients [17]. At the beginning of March 2020, Omaolo oirearvio was adopted in 79 municipalities, including the largest cities in Finland. In 2020, the use by health care provider organizations was increased to cover 69% of the Finnish population. The average number of patient users was 120,000 per week from March to September 2020. A total of 1,937,469 responses were recorded into the most frequently used Omaolo COVID-19 symptom checker in 2020 [18].

The second symptom checker, called Klinik Access, is a generic tool with a visualization of human body on the starting page. The patient first chooses the locus of the symptom and then proceeds with the reductionary dynamic form, which adapts the selections on each page for spawning the next set of possible responses onto the next page. Health care professionals receive all information gathered from the patient and an inquiry summary including preliminary diagnoses and urgency estimates. For the health care provider organizations, the tool allows symptom checking and urgency assessment to prioritize patient care [5]. The tool was adopted in 26 municipalities and private health care provider organizations in 2016-2019. The average number of patient users was 33,000 per week in 2020.

### Questionnaire

Two earlier surveys were used as models for the current study. The first identified health care professionals’ expectations of the patient portal Omaolo, which also includes oirearvio symptom checker [19]. The second survey focused on the health care professionals’ first experiences with Omaolo oirearvio [20]. In this new version of the questionnaire, health care professionals were asked to evaluate their experiences with Omaolo oirearvio or Klinik Access symptom checkers (Multimedia Appendix 2).

The questionnaire included existing validated survey items for measuring health care professionals’ support for the symptom checkers [21-23], their usability [24], and their influence on professional autonomy [25]. In addition, participants were asked to rate whether the symptom checkers had brought the planned benefits to their work and to patients. The use of these measures was piloted in the previous survey studies [14,20]. Two statements were made regarding professionals’ endorsement of the tool to patients: how often they had (1) recommended the use of and (2) instructed patients in the use of the symptom checker. For analysis, these variables were recoded into binary variables (never or at least once). Moreover, 2 open-ended questions were posed in relation to the benefits and challenges brought about by the symptom checkers to respondent’s unit or own work, which are as follows. (1) In your opinion, what challenges does the symptom checker bring to your unit or to your work? (2) In your opinion, what benefits does the symptom checker provide to your unit or your own work? To elicit background information, participants were asked about their age, gender, profession, how often they had used the symptom checkers during the previous month, and whether they had participated in the planning of the symptom checkers.

### Data Gathering

Data were gathered from February to September 2020 after the symptom checkers had been used in the 36 health organizations from 10 to 32 months. The project manager of each of the organizations sent the questionnaire link via email to the health care professionals who used Omaolo oirearvio. A manager of Klinik Healthcare Solutions, which developed Klinik Access, sent the survey invitation to the health care organizations using it. To encourage participation, 50 movie tickets for Oirearvio users and 3 tablet computers for Klinik Access users were raffled off.

### Ethics Approval

The study protocol was reviewed and approved by Aalto University Research Ethics Committee (reference 95_03.04_2019_DigiIN).

### Data Analysis

Statistical analysis was performed for the quantitative data. Descriptive statistics and reliability analyses were performed for all study variables (Multimedia Appendix 3). The Cronbach alpha scores for the scales were all above .75 indicating good internal consistency [26]. We fitted an ordinary least squares regression with robust standard errors to study the association of key independent variables, namely, benefits to professionals’ work, threat to autonomy, benefits to patients, usability, and organizational support for use with the dependent variable, namely, professionals’ support for the symptom checker. First, we fitted the univariate analyses for each independent variable to check its association with the dependent variable. Second, a model was formulated with all key variables as independent variables. Third, we added adjustments for age, gender, the symptom checker solution used, profession, participation in planning, and frequency of use. In addition, logistic regression models were fitted to study the association of the professionals’ support for the symptom checker with their promotion of it to patients and their instruction of patients in its use. These models were also adjusted for age, gender, the symptom checker solution used, profession, participation in planning, and frequency of use. In all analyses, *P* values below the .05 threshold were considered significant. When fitting the multivariate models, independent variables were added simultaneously. To test for multicollinearity, we calculated the variance inflation factors for independent variables. They were all below 2.5, indicating that multicollinearity was not a concern in this study [27].

Qualitative data were content analyzed using Atlas.ti analysis tool. Open coding was used to identify themes in the data without predefined categories. Using in vivo coding, the respondents’ words were used to define the themes to ensure that they represent the original intended meaning of the respondents.

## Results

### Respondents

The characteristics of the respondents are presented in Table 1. The respondents well represented Finnish health care professionals in terms of age, gender, and profession [28]. For example, the mean age in our sample was 42.7 years (43.0 in eligible population in Finland), and the proportion of female participants was 90.7% (88.0% in Finland). Doctors were underrepresented in comparison to nurses; in our sample, there was a little more than 1 doctor per 10 nurses, while in 2014, this number was 2.5 doctors per 10 nurses in Finland. This may be due to the large number of nurses as direct users of the symptom checkers.

**Table 1:** Respondent characteristics (n=639).

Characteristics		Values
Age (years), mean (SD)		42.7 (11.5)
**Gender, n (%)**		
	Woman	577 (90.3)
	Man	41 (6.4)
	Other or not reported	21 (3.3)
**Profession, n (%)**		
	Nurse, public nurse, or practical nurse	477 (74.7)
	Doctor	54 (8.5)
	Physiotherapist	41 (6.4)
	Other	67 (10.5)
**Participated in planning, n (%)**		
	No	568 (88.9)
	Yes	58 (9.1)
	Does not know or not reported	13 (2)
**Has provided feedback, n (%)**		
	No	400 (62.6)
	Yes	225 (35.2)
	Does not know or not reported	14 (2.2)
**Frequency of use, n (%)**		
	Every day during the last month	209 (32.7)
	Every week during the last month	244 (38.2)
	1-2 times during the last month	97 (15.2)
	Less than monthly but have tried	75 (11.7)
	Never used	14 (2.2)

### Factors Influencing Professionals’ Support for a Symptom Checker

Table 2 presents the results of the linear regression analyses testing the association of independent variables with professionals’ support for the symptom checkers. In the univariate analysis (not shown in the table), all key variables were associated with professionals’ support. All associations were positive, except for the association of threat to autonomy with support, which was negative. When added to a multivariate model (Model A), the associations of benefits to professionals’ work, usability, and benefits to patients with professionals’ support for the symptom checker remain, while the associations of organizational support for use and threat to autonomy disappear.

The independent variables account for 52% of the variation in support. The statistically significant association of benefits to patients with support disappears when adjustments for age, gender, profession, participation in planning, and frequency of use (Model B) are made. The associations of benefits to professionals’ work and usability with support remain after adjustments. Thus, the results support hypotheses 1 and 2, but only partially support hypothesis 3. No support was found for hypotheses 4 and 5. Age, gender, and participation in planning were not associated with professionals’ support.

**Table 2:** Regression model results—association of independent variables with professionals’ support. Robust standard errors were used. Continuous variables were used as continuous standardized variables.

Variable		Model A			Model B			
		*ß* (SE)	*P* value		*ß* (SE)		*P* value	
Benefits to professionals’ work		.37 (.06)	<.001		.39 (.07)		<.001	
Threat to autonomy		–.02 (.03)	.46		–.03 (.03)		.34	
Benefits to patients		.08 (.04)	.04		.06 (.04)		.16	
Usability		.27 (.05)	<.001		.27 (.05)		<.001	
Organizational support for use		.02 (.03)	.55		.02 (.03)		.52	
Age		N/A^a^	N/A		.00 (.00)		.18	
Gender (category reference: woman)		N/A	N/A		–.06 (.10)		.55	
Solution (category reference: Klinik)		N/A	N/A		–.02 (.08)		.81	
**Profession (category reference: nurse, midwife, or public health nurse)**		N/A	N/A		N/A		N/A	
	Doctor	N/A	N/A		.27 (.09)		.004	
	Physiotherapist	N/A	N/A		.09 (.12)		.44	
	Other	N/A	N/A		–.09 (.11)		.40	
Participated in planning (category reference: yes)		N/A	N/A		–.06 (.11)		.56	
**Frequency of use (category reference: every day during the last month)**		N/A	N/A		N/A		N/A	
	Every week during the last month			N/A		–13 (.07)		.047
	1-2 times during the last month	N/A	N/A		–.10 (.09)		.27	
	Less than monthly but have tried	N/A	N/A		–.06 (.10)		.54	
	Never used	N/A	N/A		–.20 (.17)		.26	
R-squared		.52	N/A		.52		N/A	

^a^N/A: not applicable.

### Association of Professionals’ Support With Their Recommendation of the Tool to Patients and Provision of Instruction Regarding Its Use

The results of the logistic regression models (Multimedia Appendices 4 and 5) show that support for the symptom checkers was associated with both professionals’ recommendation to the patients and their provision of instruction to patients regarding use of the symptom checkers. The associations were maintained even when adjustments for gender, age, profession, symptom checker solution, and participation in the planning of the solution were made. Hypotheses 6 and 7 were thus supported.

### Perceived Benefits and Disadvantages of the Symptom Checkers

Of the total 639 health care professionals, 216 (33.8%) responded to the open-ended questions. Most of the health care professionals (164/216, 75.9%) reported both benefits and disadvantages of the symptom checkers, 30/216 (13.9%) perceived only disadvantages, and 22/216 (10.2%) perceived only benefits.

Table 3 summarizes the benefits of the symptom checkers that health care professionals described in their responses to the open-ended questions. The symptom checkers were perceived to be beneficial for work, as they streamline the work in various ways, such as by providing preliminary information on patients and by decreasing telephone work. Patients were able to receive help expeditiously by using symptom checkers. The symptom checkers were considered to lower the threshold for care, and the self-care instructions were perceived useful for patients.

The most frequently mentioned challenges were related to the characteristics and use of the symptom checkers (Table 4). The results of the assessment provided by the symptom checkers were perceived to be inaccurate and unfocused, so the professionals still needed to call patients and ask clarifying questions.

Users of the symptom checkers found it challenging to use the services alongside other work tasks, as switching between tasks is troublesome. Patients contacted them using several channels, which were a symptom checker, calling, and visiting. Evidently, the symptom checkers did not clearly inform patients how a health care professional will contact them.

Many also mentioned that patients cannot use the symptom checker, or that they do not understand the questions or wordings. Health care professionals were also concerned that older patients are unable to use the service. They suggested that the services should be advertised more, as patient usage is low.

**Table 3:** Perceived benefits of the symptom checkers evaluated.

Themes			Mentions, n (%)
**Facilitates health care professionals’ work**			
	Streamlines work by providing preliminary information on patients	75 (11.7)	
	Reduces the number of phone calls	52 (8.1)	
	Makes work flexible, a patient case can be handled at a suitable time slot	27 (4.2)	
	Comprehensive preliminary information on patients	12 (1.9)	
	Patients’ own descriptions of the symptoms can be used in medical reports	5 (0.8)	
	Fluent communication with patients	5 (0.8)	
	Gives variety to the work	5 (0.8)	
	Reduce the number of visits	5 (0.8)	
**The symptom checker is beneficial for patients**			
	Patients receive help easily and quickly	49 (7.7)	
	Supports self-management	22 (3.4)	
	Lowers threshold for care	20 (3.1)	
	Urinary tract infection and sexually transmitted disease symptom checkers are especially useful	19 (3.0)	
	Uniforms quality of triage	18 (2.8)	
	Useful during the COVID-19 pandemic	8 (1.3)	

**Table 4:** Perceived disadvantages of the symptom checkers evaluated.

Themes		Mentions, n (%)
**The symptom checker is not working in an optimal way**		
	Communicating with patients is time-consuming or cumbersome	74 (11.6)
	There is a need to call patients and ask clarifying questions	56 (8.8)
	Provides inaccurate results	54 (8.5)
	Cumbersome to use or it should be more automatic	49 (7.7)
	Not interoperable with patient health records	36 (5.6)
	Too sensitive	19 (3.0)
	Provides a poor summary; it is difficult to identify essentials	16 (2.5)
	It is easier and faster to evaluate a patient’s condition (eg, breathing over the phone)	15 (2.3)
	Appointments are challenging to make via the symptom checker	14 (2.2)
	Signing in repeatedly is slow	13 (2.0)
**The workflow with the symptom checker is not optimal**		
	Is included among many other tasks, and managing time between different tasks is challenging	66 (10.3)
	Creates extra work or slows down working	53 (8.3)
	The method for organizing work is unclear; a commonly agreed course of action for responding to patients is missing	17 (2.7)
	Patients contact using several channels	31 (4.9)
**Patients have difficulties with the symptom checker**		
	Not all patients are able to use symptom checkers (eg, older people)	33 (5.2)
	Few patients use symptom checkers; there should be more advertising	30 (4.7)
	Patients do not know how to use the symptom checker	14 (2.2)
	Patients do not understand all the questions or wordings of the service	10 (1.6)
	It is not clear for patients how professionals contact them	2 (0.3)
	Health care professionals need more experience or do not know how to use the symptom checker	8 (1.3)
	Resistance to change	7 (1.1)

## Discussion

### Principal Findings

The results indicate that in the sustained use of the symptom checkers for triage, benefits to health care professionals’ work, and usability were the most important factors influencing professionals’ support for the tool. Benefits to patients were also positively associated with health care professionals’ support for symptom checkers as has previously been found in the implementation phase [14]. However, the association weakened when the control variables were added to the model.

Organizational support for use was positively associated, and threat to professionals’ autonomy was negatively associated with health care professionals’ support. These associations were, however, not independent of professionals’ perceptions of usability and benefits to work. Organizational support may have already been incorporated to the perception of the technology’s usability and benefits in work [29].

The open-ended question responses of the professionals deepen understanding of the underlying reasons for the associations found in the quantitative analysis. The influences on professionals’ work were seen both positive and negative; the tools streamlined work by providing preliminary information on patients and reduced the number of phone calls, but they also created extra work as the professionals needed to call patients and ask clarifying questions. The need to ask more questions arose from the experienced inaccuracy of the symptom checkers and unclear assessment reports. Health care professionals also reported that patients do not always know how to use symptom checkers, or they may not understand all the wordings of the service.

Based on the health care professionals’ experiences, symptom checkers are beneficial for patients as they receive help quickly with lower threshold. Moreover, the services support self-management. The experienced benefits of symptom checkers of sensitive diseases, such as sexually transmitted diseases, suggest that one of the potentials of the symptom checkers may be to lower the threshold for care in such cases, as suggested by Johansen et al [30]. The perceived uniform quality of triage is also beneficial to patients as all essential questions are asked regardless of the skill level of the health care professional. Symptom checkers were perceived to be particularly beneficial during the COVID-19 epidemic. In fact, during the second wave, 1,550,000 people used the Omaolo COVID-19 symptom checker [18].

### Comparison With Prior Work

Consistent with earlier studies that focused on professionals’ attitudes, usability and utility were the most common factors in promoting adoption by health care professionals [14,31,32]. For symptom checkers, their effective utility in professionals’ work was critical. This is in line with earlier findings that the fit between clinical work tasks and the technology design has a significant influence on the adoption of health innovations [10,33]. In agreement with our results, Entezarjou et al [34] found that automated patient interview can streamline clinical work. However, our results support an earlier interpretation that intelligent triage tools may also increase professionals’ workload, as the information provided by patients via these tools entails gaps and uncertainty in data [35].

In line with this study, Cajander et al [36] found that digital communication with patients may in some cases slow down the assessment of care need. However, in their study, nurses found digital communication to be emotionally less stressful than phone calls. Better care for patients has been found to be an important benefit of eHealth services from professionals’ [34,37] and leaders’ point of view [38]; nonetheless, in the acceptance models, benefits to patients have been overlooked [39].

Professionals’ experience of the inaccuracy of the assessment reports may have emerged from many sources, such as how they perceived patients’ use of symptom checkers. As Marco-Ruiz et al [3] mentioned, the accuracy of the symptom checkers depends on how well patients are able to communicate their symptoms with the tools. Based on their interview study, Tsai et al [40] found that patients sought explanations for the results obtained from web-based symptom checkers. They showed that better explanations and more transparent results improved patient trust on the diagnostic quality of the results and helped them come up with better decisions.

### Limitations

The limitations of this study are related to the cross-sectional, single-informant design, as well as the omission of likely relevant contextual factors in the models. First, this study relied on professionals’ self-reported perceptions of symptom checkers. The single-informant design may lead to inflation of the strength of relationships. To mitigate this problem, the measures validated in previous studies were applied. Second, in this study, we were not able to perform a longitudinal analysis of the implementation of the symptom checkers in the organizations. To address the dynamic nature of information technology implementation, we compared our findings with professionals’ perceptions of another eHealth tool studied in the preimplementation phase in Finland. The findings allowed us to come up with preliminary suggestions on which factors explain professionals’ support for an eHealth tool in the preimplementation phase as opposed to the use phase. Third, to limit the length of the questionnaire, we were not able to include all relevant contextual factors in our analysis. The factors that may offer support for symptom checkers are, for instance, the competence of the professionals in using these tools [13,41] and vision for the future development of the tools [19], among others. However, as various symptom checkers are used across many health care contexts and numerous purposes, the research results may not be generalizable to other settings.

### Future Directions for Research and Practice

The findings suggest that more research is needed on the successful blended use of digital and traditional communication channels by professionals in triage. In addition, many health care professionals were concerned that not all patients, particularly the older people, are able to use symptom checkers; usage statistics confirm that 20- to 29-year-olds were the largest user group [18]. Thus, future studies are needed to support the wider adoption of symptom checkers and health equity. Furthermore, the transparency and explainability of symptom checkers are worth studying in the future from health care professionals’ point of view.

The results imply that it is imperative to develop symptom checkers that are usable and support health care professionals’ work. Furthermore, health organizations and teams need to carefully reorganize the work processes and distribution of work so that the use of symptom checkers is smoothly integrated among other tasks. For example, the phone and digital work can be allocated to health care professionals every other week. In addition, health organizations need to ensure that patients are well instructed, are aware that the professionals will contact them, and know that they do not need to initiate contact using several channels simultaneously.

While this research did not investigate the specific tasks different health care professionals perform using the symptom checkers, it is likely that the tasks in the initial and more specialized screening of patient inquiries differ between professional groups. To better understand the potential of symptom checkers to supplement resource-intensive telephone triage lines common in primary care, more research on division of tasks between different professional groups in symptom checker–supported triage is needed.

## Appendix

Multimedia Appendix 1 Description of symptom checkers.

Multimedia Appendix 2 Questionnaire.

Multimedia Appendix 3 The means, standard deviations, and Cronbach alphas of key study variables.

Multimedia Appendix 4 Logistic regression results; predictors of promotion of the symptom checker to the patients.

Multimedia Appendix 5 Logistic regression results; predictors of instructing patients in the use of the symptom checker.
