# Supplementary material for: Health Care Professionals’ Experiences of Web-Based Symptom Checkers for Triage: Cross-sectional Survey Study
Source: J Med Internet Res. 2022 May 5;24(5):e33505. doi: 10.2196/33505 (PMC9121216; doi:10.2196/33505)

**Multimedia Appendix 1**

**Description of symptom checkers.** (Adapted from CONSORT guideline extension for evaluation of interventions with an AI component, CONSORT-AI)

|  | Omaolo | Klinik Access |
| --- | --- | --- |
| CE | CE MDD Class I marked since 3/2020. EBMEDS CE Class I marked since 9/2014 | CE MDD Class I marked since 2018. |
| AI/Algorithm | The Evidence-Based Medicine Electronic Decision support (EBMEDS^®^) system combines electronic patient record data with medical data based on algorithms. | A static probabilistic model that generates list of possibilities and infers urgency. The algorithm is the third evolution for the company which includes the severity signs model and state-of-art likelihood-ratio based probability calculations. |
| Input | First, patients select the symptom checker suitable to their condition or a generic checker. Second, they fill in a dynamic and adaptive questionnaire form and describe symptoms. Many symptom checkers include also free text fields. | The medical algorithm generates a reductionary dynamic form, which adapts the patient selections on each page for spawning the next set of possible responses onto the next page. The dynamic form produces free text questions in addition to parametric selections. |
| Poor quality handling | Algorithms include functions that aim to minimize or eliminate the effects of the poor-quality input. | Variations in patient selections is managed by clinical curation of usability and trustworthiness of the inputs, which is taken into account in correlation strengths accordingly following clinical practice and experience. |
| Output | The health professional receives a report of the patient’s symptoms, the most probable diagnoses, and the quality and urgency of the care needed. The patient receives self-care instructions or request to contact a health professional. Depending on the locality, patient can reserve an appointment or send the assessment results to a health professional. | All information that is input by the patient is output into the inquiry Summary for the health professional, along with the differential and urgency estimates suggested by the algorithm. The solution directs the patient to contact health center or emergency care if the inquiry is flagged as a potential emergency (highest rating). |
| Clinical practice | The healthcare professionals go through all inquiries in their dashboard. Patient inquiries are redirected to different units based on configuration and the selections that were made by the patient. Healthcare staff can organise inquiry management as they wish. The professional makes their own informed decision on triage actions, and notify the patient accordingly. | The health professionals go through all inquiries in their interface. Patient inquiries are redirected to different units based on configuration and the selections that were made by the patient. Healthcare staff can organise inquiry management as they wish, typically by prioritising clinical inquiries to nurses or doctors and by going through the list based on suggested the priority from the algorithm. The staff member makes their own informed decision on triage actions, and notify the patient accordingly. |

The Omaolo oirearvio user interface


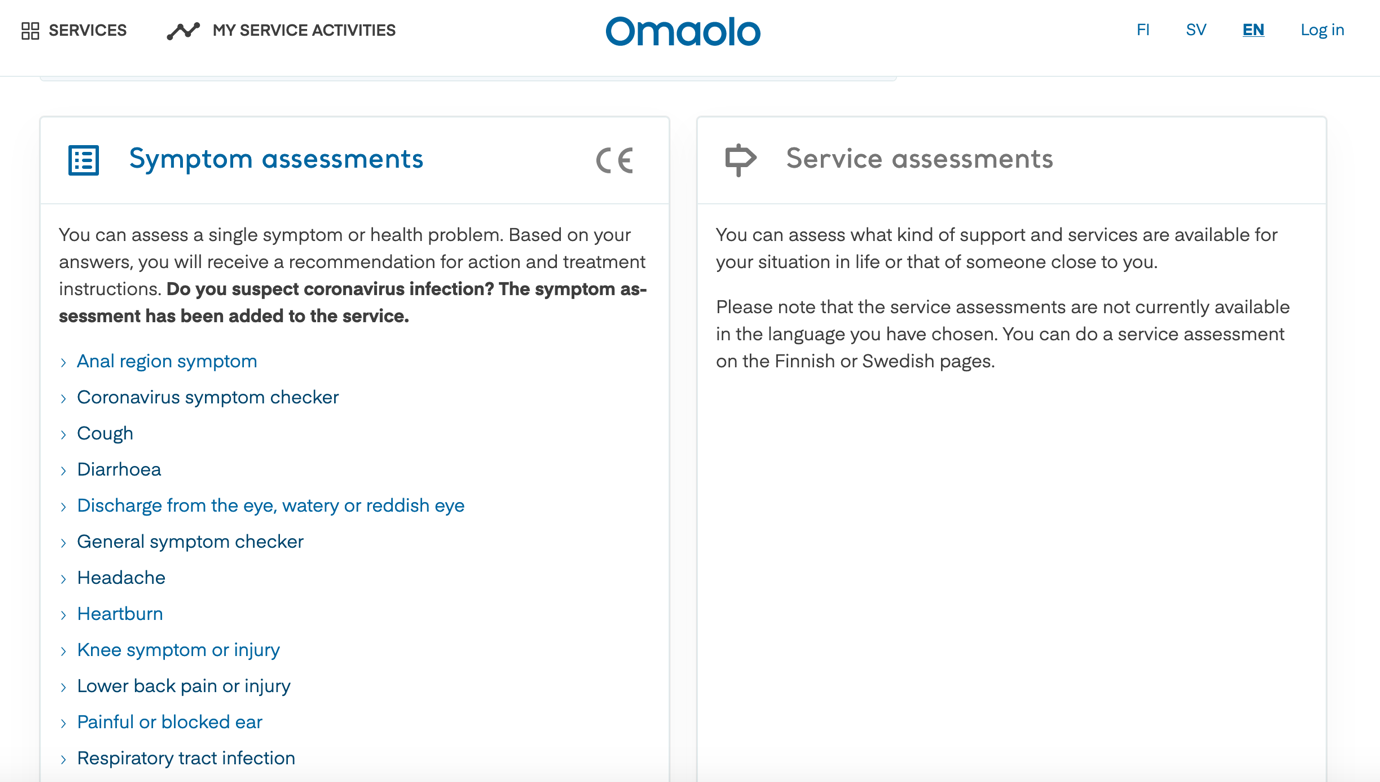


The Klinic Acces user interface for patients


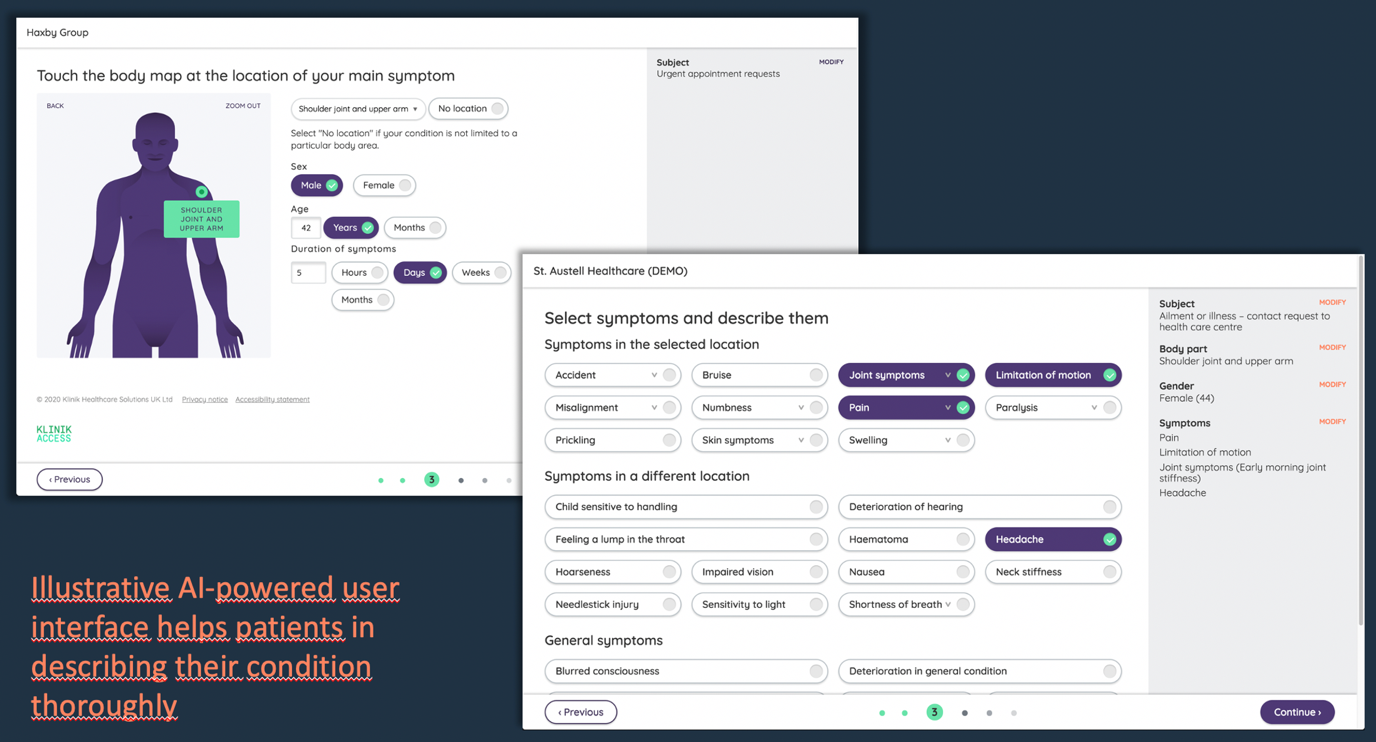


The Klinic Acces user interface for health professionals


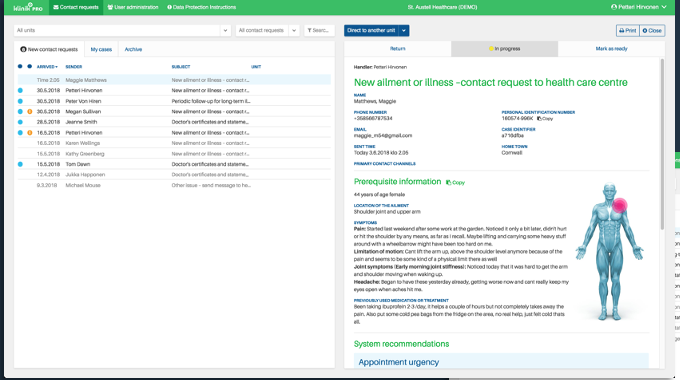

Supplement: Multimedia Appendix 1 [file jmir_v24i5e33505_app1.docx]
