# Supplementary material for: Health Care Professionals’ Experiences of Web-Based Symptom Checkers for Triage: Cross-sectional Survey Study
Source: J Med Internet Res. 2022 May 5;24(5):e33505. doi: 10.2196/33505 (PMC9121216; doi:10.2196/33505)
Supplement: Multimedia Appendix 2 [file jmir_v24i5e33505_app2.docx]

**Multimedia Appendix 2**

**Questionnaire**

The scales ranged from 1 (strongly disagree) to 5 (strongly agree) and included option 6 (I don’t know).

**SUPPORT FOR THE SYMPTOM CHECKER**

What is your opinion about the use of the symptom checker?

1. I support the use of the symptom checker.
2. My co-workers support the use of the symptom checker.
3. My supervisor supports the use of the symptom checker.
4. I understand the organization’s decision to implement the symptom checker.

**INFLUENCES ON PROFESSIONALS’ WORK**

Please evaluate the influence of the symptom checker on your work:

1. The symptom checker speeds up my tasks.
2. The symptom checker provides me with more useful information about a patient.
3. The symptom checker creates uniformity in the quality of the triage.
4. The symptom checker increases the attractiveness of the professionals’ work.

**THREAT TO PROFESSIONAL AUTONOMY**

Please evaluate the influence of the symptom checker to your professional role:

1. The symptom checker will decrease my control over the patient care process.
2. The symptom checker will decrease my control over professional decisions.

**BENEFITS TO PATIENTS**

Please evaluate the influence of the symptom checker on patients in your unit:

1. By using the symptom checker, patients are able to receive help quickly.
2. I believe that more than half of the patients are willing to use the symptom checker.
3. I believe that more than half of the patients are capable of using the symptom checker.
4. Based on my experience, patients understand the advice given by the symptom checker.

**PROMOTION OF THE SYMPTOM CHECKER TO PATIENTS** (Options: Never, 1–3 times, 4 times or more)

I have recommended patients to use the symptom checker.

**INSTRUCTION OF THE PATIENTS IN THE USE OF THE SYMPTOM CHECKER** (Options: Never, 1–3 times, 4 times or more)

I have advised patients in using the symptom checker.

**CHALLENGES AND BENEFITS**

In your opinion, what challenges do the symptom checker bring to your unit or to your work? (Open-ended question)

In your opinion, what benefits do the symptom checker provide to your unit or your own work? (Open-ended question)

**USABILITY**

Evaluate what is it like to use the symptom checker in your work?

1. The symptom checker meets my requirements.
2. Using the symptom checker is a frustrating experience.
3. The symptom checker is easy to use.

**ORGANIZATIONAL SUPPORT FOR USE**

Have you received support for using the professional part of the symptom checker?

1. I have received enough information about the benefits of the symptom checker.
2. I have been encouraged to use the symptom checker.
3. I have received enough orientation to use the symptom checker.
4. I have received enough information on how to give feedback on the symptom checker.
5. I have received enough help if there are problems encountered in using the symptom checker.

**What kind of support, information or training would you like to have in relation to the symptom checker?** (Open-ended question)

**How often did you use the symptom checker in the previous month?**

(Options: Daily, Weekly, 1 or 2 times a month, Less frequently/I have tried it, Never used it)
