# Supplementary material for: Health Care Professionals’ Experiences of Web-Based Symptom Checkers for Triage: Cross-sectional Survey Study
Source: J Med Internet Res. 2022 May 5;24(5):e33505. doi: 10.2196/33505 (PMC9121216; doi:10.2196/33505)
Supplement: Multimedia Appendix 3 [file jmir_v24i5e33505_app3.docx]

**Multimedia Appendix 3**

The means, standard deviations and Cronbach alphas of key study variables (n=637)

| Variable | N | Mean | SD | Cronbach alpha |
| --- | --- | --- | --- | --- |
| Support | 638 | 4.1 | 0.92 | .87 |
| Benefits for professionals' work | 637 | 3.2 | 1.07 | .89 |
| Threat to autonomy | 613 | 2.5 | 1.00 | .85* |
| Benefits for patients | 637 | 3.4 | 0.95 | .80 |
| Usability | 635 | 3.7 | 0.93 | .76 |
| Organizational support for use | 635 | 3.6 | 0.96 | .87 |
| The measurement scales were ranging from 1 (fully disagree) to 5 (fully agree) with additional option 6 (I don’t know) for all variables. | | | | |
| *Scale reliability coefficient is reported for this two-item scale | | | |  |
