# Supplementary material for: Health Care Professionals’ Experiences of Web-Based Symptom Checkers for Triage: Cross-sectional Survey Study
Source: J Med Internet Res. 2022 May 5;24(5):e33505. doi: 10.2196/33505 (PMC9121216; doi:10.2196/33505)
Supplement: Multimedia Appendix 4 [file jmir_v24i5e33505_app4.docx]

**Multimedia Appendix 4**

Logistic regression results - Predictors of promotion of the symptom checker to the patients.

|  |  | Model A | | |  | Model B | | |
| --- | --- | --- | --- | --- | --- | --- | --- | --- |
|  |  | OR | 95% CI | *P* value |  | OR | 95% CI | *P* value |
| Support for the symptom checker | | 1.87 | 1.56–2.24 | <.001 |  | 2.10 | 1.70–2.61 | <.001 |
| Age |  |  |  |  |  | 1.03 | 1.01–1.06 | .001 |
| Gender | |  |  |  |  |  |  |  |
|  | Woman |  |  |  |  | 1 |  |  |
|  | Man |  |  |  |  | 0.82 | 0.35–1.92 | .64 |
| Solution | |  |  |  |  |  |  |  |
|  | Klinik |  |  |  |  | 1 |  |  |
|  | Omaolo |  |  |  |  | 0.68 | 0.40–1.14 | .142 |
| Profession | |  |  |  |  |  |  |  |
|  | Nurse/midwife/public health nurse |  |  |  |  | 1 |  |  |
|  | Doctor |  |  |  |  | 0.23 | 0.11–0.50 | <.001 |
|  | Physiotherapist |  |  |  |  | 0.38 | 0.17–0.85 | .018 |
|  | Other |  |  |  |  | 0.32 | 0.16–0.65 | .002 |
| Participated in planning | |  |  |  |  |  |  |  |
|  | Yes |  |  |  |  | 1 |  |  |
|  | No |  |  |  |  | 1.76 | 0.78–4.01 | .175 |
| Frequency of use | |  |  |  |  |  |  |  |
|  | Every day during the last month |  |  |  |  | 1 |  |  |
|  | Every week during the last month |  |  |  |  | .72 | 0.40–1.31 | .282 |
|  | 1-2 times during the last month |  |  |  |  | .38 | 0.19–0.77 | .007 |
|  | Less than monthly but have tried |  |  |  |  | .18 | 0.09–0.38 | <.001 |
|  | Have never used |  |  |  |  | .05 | 0.01–0.24 | <.001 |
| Note: Continuous variables were used as continuous standardized variables. | | | | | | |  |  |
