# Supplementary material for: Health Care Professionals’ Experiences of Web-Based Symptom Checkers for Triage: Cross-sectional Survey Study
Source: J Med Internet Res. 2022 May 5;24(5):e33505. doi: 10.2196/33505 (PMC9121216; doi:10.2196/33505)
Supplement: Multimedia Appendix 5 [file jmir_v24i5e33505_app5.docx]

**Multimedia Appendix 5**

Logistic regression results - Predictors of instructing patients in the use of the symptom checker.

|  |  | Model A | | |  | Model B | | |
| --- | --- | --- | --- | --- | --- | --- | --- | --- |
|  |  | OR | 95% CI | *P* value |  | OR | 95% CI | *P* value |
| Support for the symptom checker | | 1.44 | 1.22–1.70 | <.001 |  | 1.42 | 1.18–1.71 | <.001 |
| Age |  |  |  |  |  | 1.01 | 1.00–1.03 | .067 |
| Gender |  |  |  |  |  |  |  |  |
|  | Woman |  |  |  |  | 1 |  |  |
|  | Man |  |  |  |  | 1.23 | 0.55–2.80 | .61 |
| Solution |  |  |  |  |  |  |  |  |
|  | Klinik |  |  |  |  | 1 |  |  |
|  | Omaolo |  |  |  |  | 0.84 | 0.57–1.25 | .40 |
| Profession | |  |  |  |  |  |  |  |
|  | Nurse/midwife/public health nurse | |  |  |  | 1 |  |  |
|  | Doctor |  |  |  |  | 0.16 | 0.06–0.38 | <.001 |
|  | Physiotherapist |  |  |  |  | 0.42 | 0.21–0.87 | .019 |
|  | Other |  |  |  |  | 0.37 | 0.20–0.69 | .002 |
| Participated in planning | |  |  |  |  |  |  |  |
|  | Yes |  |  |  |  | 1 |  |  |
|  | No |  |  |  |  | 2.80 | 1.43–5.46 | .003 |
| Frequency of use | |  |  |  |  |  |  |  |
|  | Every day during the last month |  |  |  |  | 1 |  |  |
|  | Every week during the last month |  |  |  |  | .73 | 0.49–1.11 | .14 |
|  | 1-2 times during the last month |  |  |  |  | .38 | 0.21–0.68 | .001 |
|  | Less than monthly but have tried |  |  |  |  | .28 | 0.15–0.53 | <.001 |
|  | Have never used |  |  |  |  | .11 | 0.02–0.63 | .013 |
| Note: Continuous variables were used as continuous standardized variables. | | | | | | |  |  |
